# The Albany Medical College

**Published:** 1871-02

**Authors:** 


### THE ALBANY MEDICAL COLLEGE.

The daily papers published in the city of Albany, some time since, contained numerous grave charges of irregularities against the Albany Medical College and its Faculty—which, if true, would have merited the universal condemnation of the entire profession. As Southern Journalists, born and reared in the South, we nevertheless have, we trust, a catholicity of feeling which, in medicine, knows no East, West, North or South. Hence we hope our Northern medical friends will not deem our allusions to their institutions out of place, or void of kindly feelings towards them. In the present instance, it was stated that a disruption of the Faculty of the Albany Medical College had occurred in consequence of discretitable irregularities on the part of the Faculty.

It was charged, among other things, that the Hon. Mr. Harris, Professor of Medical Jurisprudence, had manifested homœpathic affinities. Mr. Harris is not properly one of the Faculty—but is simply a lecturer upon Medical Jurisprudence. Yet, so necessary is it for the Profession to maintain its purity, that even his connection with a regular college, with such affinities, would be damaging to the cause of Medical Science—being a violation of the principles controlling the Profession. We are glad to learn, however, that the charges are false, and that the Faculty of the College, including Mr. Harris, have made no departures from the true Ethics of the Profession. It affords us pleasure to make such an announcement. But the Ethical points involved in the discussion of the reported irregularities of the Albany Medical College and its Faculty, affords a case in point of the importance and necessity of our medical institutions maintaining the principles upon which the Profession are determined shall control and govern its recognized Schools. They have a right to say what these principles shall be; and while no coercive action can be taken by them, to enforce upon medical institutions the principles upon which they shall be controlled, yet they must and will deny them, when refractory and irregular, that patronage from which they draw their support, and by refusing to recognize their students or diplomas. The attempt to run a medical school upon an independent line, will inevitably result in disaster to all connected with it. The charter of a College may be legal, just as in the case of the Albany Medical College, but if any one connected with such an institution, either of its Faculty or its Board of Trustees, should attempt to conduct it contrary to the laws controlling the profession and the terms of the charter, it will then become irregular and illegal. It is legal for Homœpathics or Eclectics to charter institutions to promulgate their peculiar ideas—but should a Board of Trustees of such a College, attempt to mix their Faculties with Regulars, Hydropaths or Hindoo jugglars, they would become illegal by such action. A Board of Trustees transcend their legal powers whenever they, at their option, disregard the terms of a College charter, whether of an orthodox or irregular institution. And when they do so—when they violate not only professional but statutory law, they place themselves as offenders against both, and justly subject themselves to the opprobrium of all law-abiding citizens, both in or out of the profession. Such an institution, with such a board of trustees, cannot hope to meet the approval, recognition, or the support of the Profession—a fact which, in such cases, as in all others of flagrant wrong, prove the truth of the assertion, "that the way of the transgressor is hard."

